# Supplementary material for: GC, GC/MS Analysis, and Biological Effects of Essential Oils from Thymus mastchina and Elettaria cardamomum
Source: Plants (Basel). 2022 Nov 23;11(23):3213. doi: 10.3390/plants11233213 (PMC9793757; doi:10.3390/plants11233213)
Supplement: Supplementary file 1 [file plants-11-03213-s001.zip › plants-2011718-supplementary.pdf]

Supplementary Materials

for

GC, GC/MS analysis and biological effects of essential oils from *Thymus mastchina* and *Elettaria cardamomum*

Nenad L. Vukovic [1*], Milena D. Vukic [1], Ana D. Obradovic [2], Milos M. Matic [2], Lucia Galovičová [3], Miroslava Kačániová [3,4]

[1] Department of Chemistry, Faculty of Science, University of Kragujevac, 34000 Kragujevac, Serbia
[2] Department of Biology and Ecology, Faculty of Science, University of Kragujevac, 34000 Kragujevac, Serbia
[3] Institute of Horticulture, Faculty of Horticulture and Landscape Engineering, Slovak University of Agriculture, Tr. A. Hlinku 2, 94976 Nitra, Slovakia
[4] Department of Bioenergy, Food Technology and Microbiology, Institute of Food Technology and Nutrition, University of Rzeszow, 4 Zelwerowicza St, 35601 Rzeszow, Poland
*Correspondence: nenad.vukovic@pmf.kg.ac.rs; Tel.: +381336223

Content

Figure S1. GC/MS chromatogram of series of n-alkanes (C_7–C_{35}) for calculation of Kovats retention indices for *T. mastichina* EO.

Figure S2. GC/MS chromatogram of *T. mastichina* EO for calculation of Kovats retention indices.

Figure S3. GC/MS chromatogram of series of n-alkanes (C_7–C_{35}) for calculation of Van Den Dool retention indices for *T. mastichina* EO.

Figure S4. GC/MS chromato-gram of *T. mastichina* EO for calculation of Van Den Dool retention indices.

Figure S5. GC/MS chromatogram of series of n-alkanes (C_7–C_{35}) for calculation of Kovats retention indices for *E. cardamomum* EO.

Figure S6. GC/MS chromatogram of *E. cardamomum* EO for calculation of Kovats retention indices.

Figure S7. GC/MS chromatogram of series of n-alkanes (C_7–C_{35}) for calculation of Van Den Dool retention indices for *E. cardamomum* EO.

Figure S8. GC/MS chromatogram of *E. cardamomum* EO for calculation of Van Den Dool retention indices.

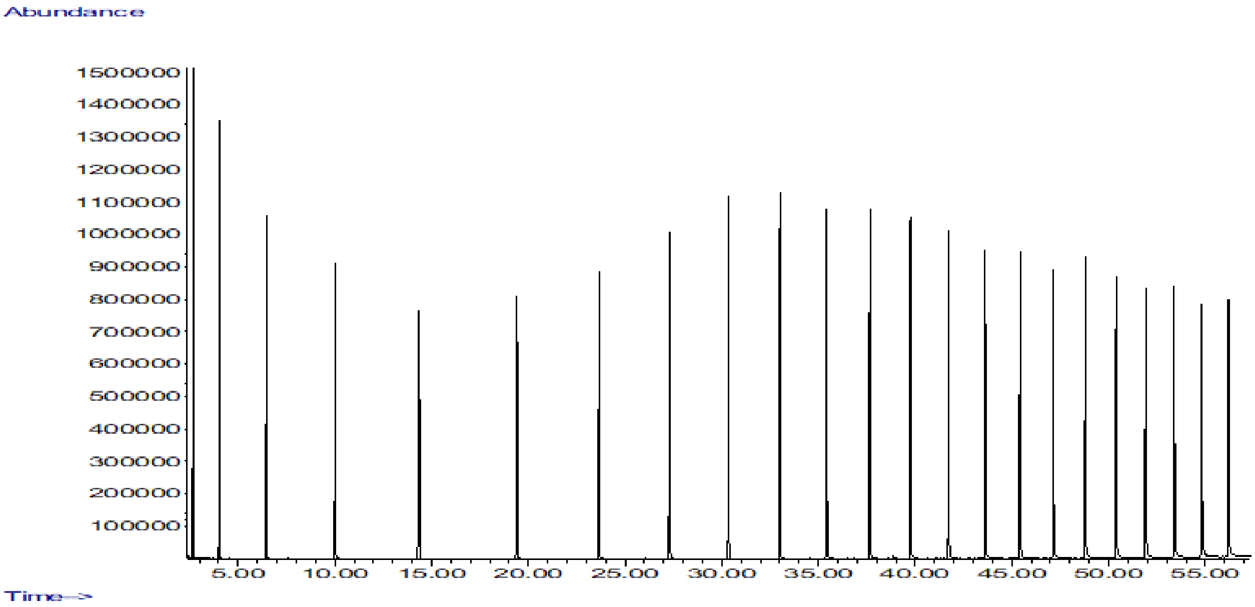

Figure S1. GC/MS chromatogram of series of n-alkanes (C_7–C_{35}) for calculation of Kovats retention indices for *T. mastichina* EO.

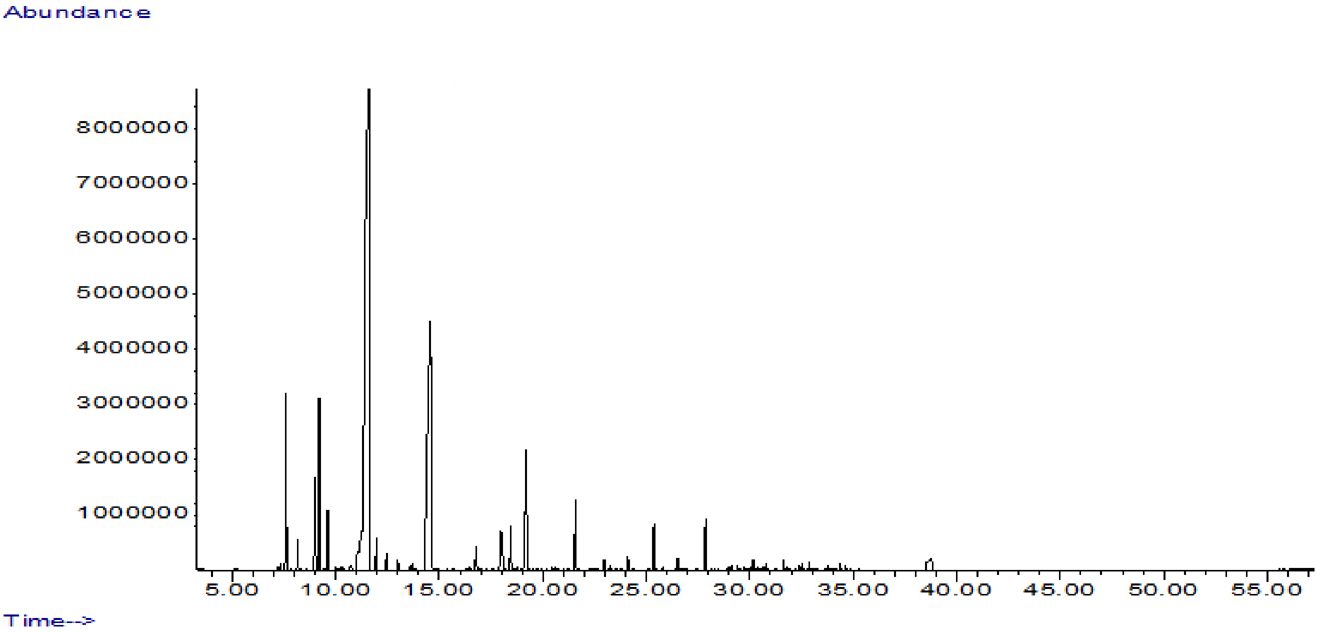

Figure S2. GC/MS chromatogram of *T. mastichina* EO for calculation of Kovats retention indices.

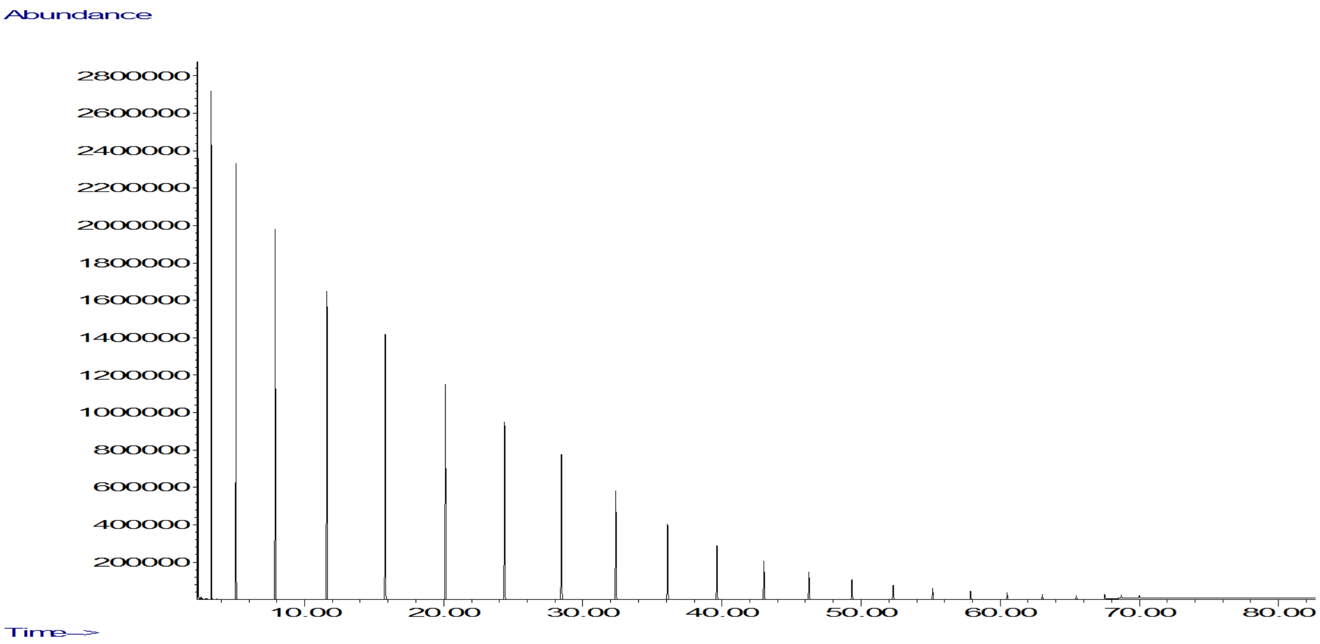

Figure S3. GC/MS chromatogram of series of n-alkanes (C_7–C_{35}) for calculation of Van Den Dool retention indices for *T. mastichina* EO.

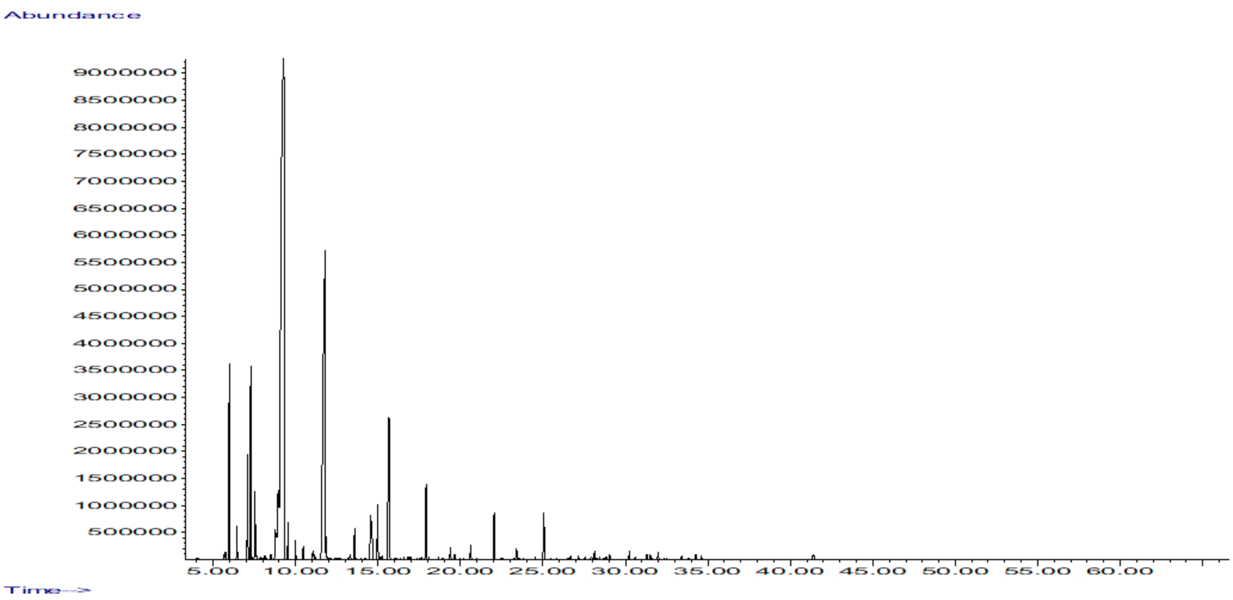

Figure S4. GC/MS chromatogram of *T. mastichina* EO for calculation of Van Den Dool retention indices.

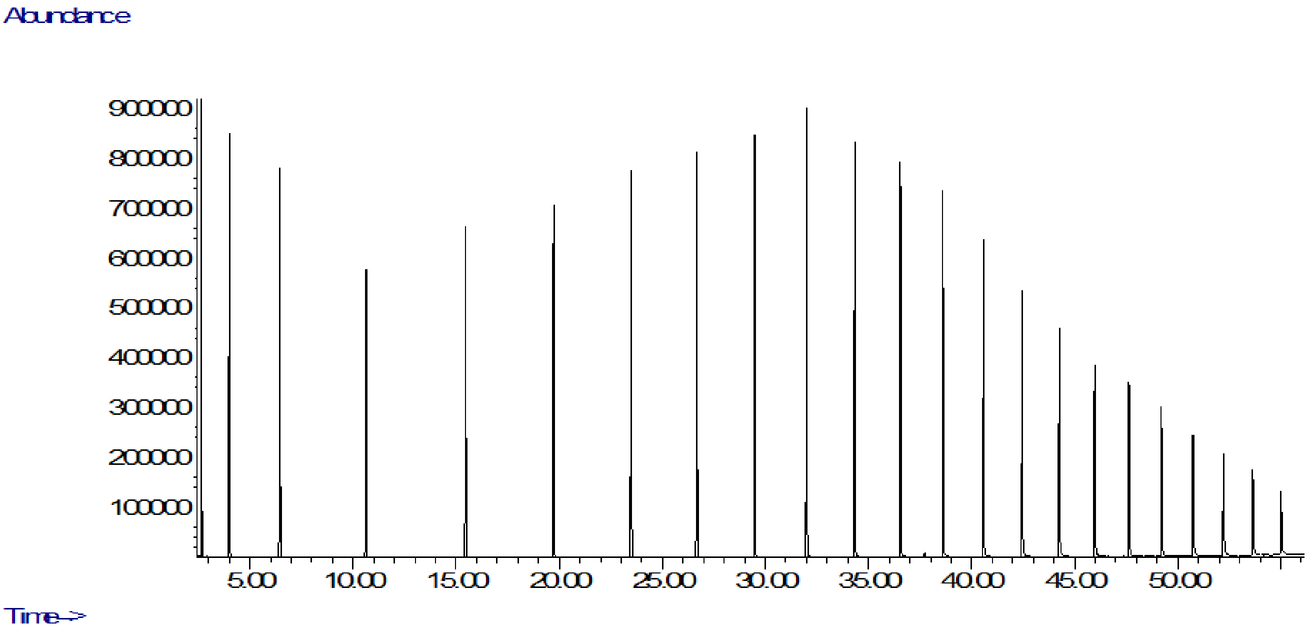

Figure S5. GC/MS chromatogram of series of n-alkanes (C_7–C_{35}) for calculation of Kovats retention indices for *E. cardamomum* EO.

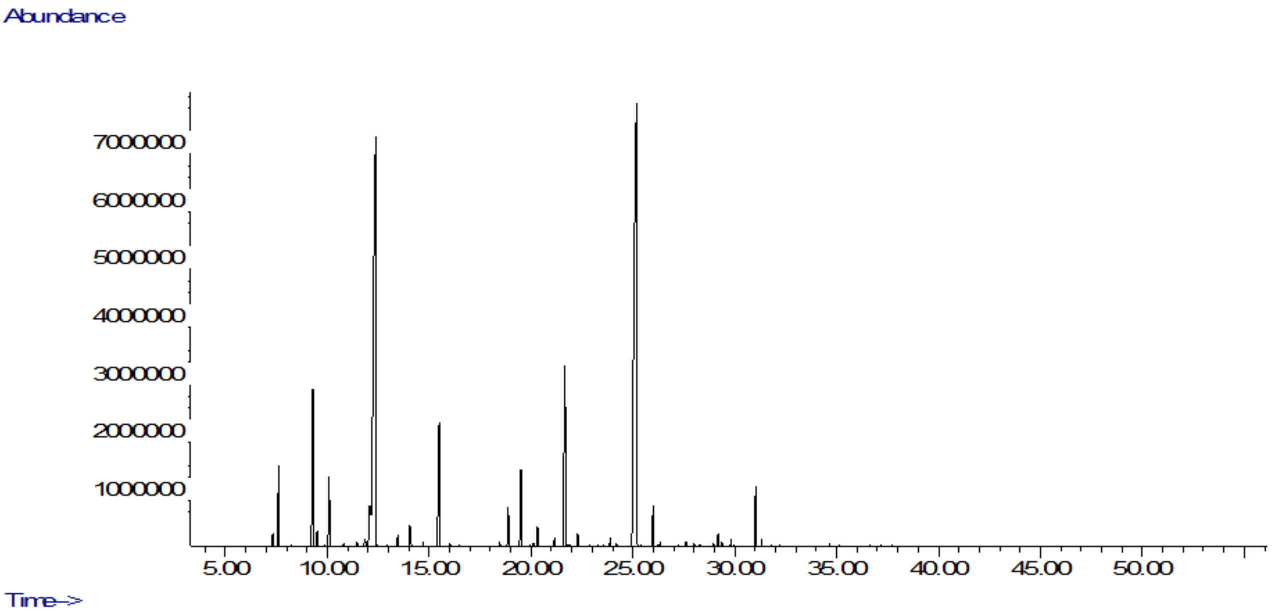

Figure S6. GC/MS chromatogram of *E. cardamomum* EO for calculation of Kovats retention indices.

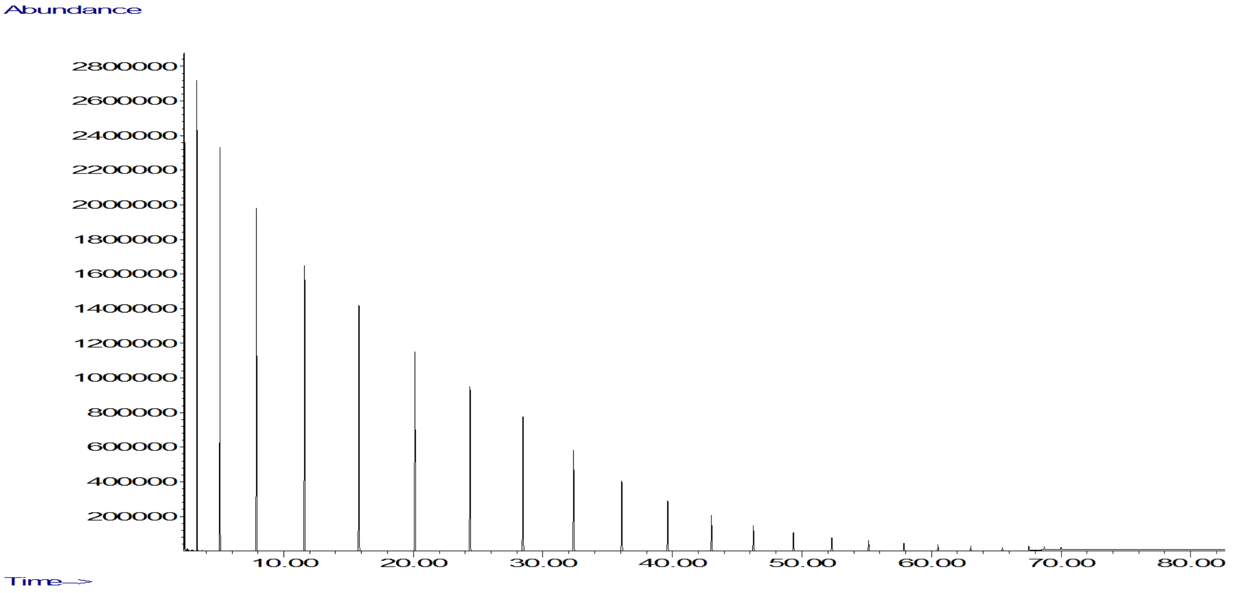

Figure S7. GC/MS chromatogram of series of n-alkanes (C_7–C_{35}) for calculation of Van Den Dool retention indices for *E. cardamomum* EO.

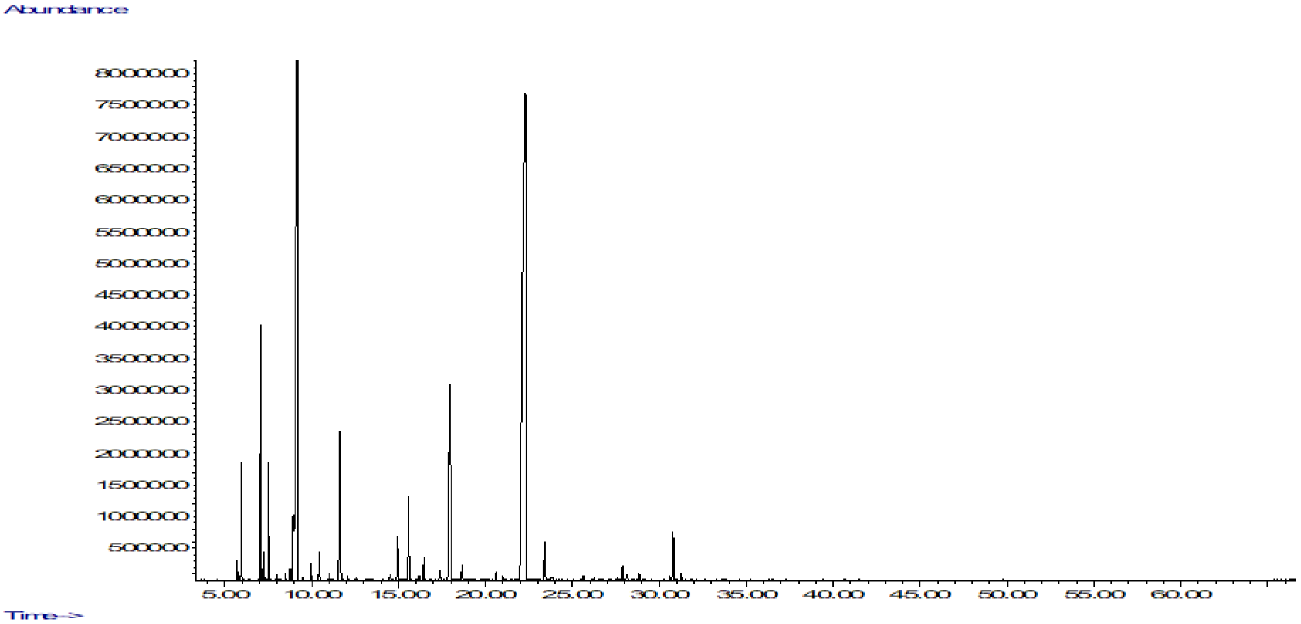

Figure S8. GC/MS chromatogram of *E. cardamomum* EO for calculation of Van Den Dool retention indices.
